# No More High Notes: A Sequel of Spontaneous Laryngeal Rupture

**DOI:** 10.7759/cureus.71965

**Published:** 2024-10-20

**Authors:** A Lezith Marroquin Rodriguez, Claudia J Rodriguez Reus, Amaury J Valdés Mancha, Edgar A Cantu Rodriguez, Adrián A Negreros-Osuna

**Affiliations:** 1 Radiology Department, Hospital Regional Institute of Security and Social Services for State Workers (ISSSTE) Monterrey, Autonomous University of Nuevo León, Monterrey, MEX

**Keywords:** laringeal, rupture, sequel, spontaneus, thyroarytenoid

## Abstract

Spontaneous laryngeal rupture is an unusual event, typically caused by a sudden increase in barometric pressure and biomechanical forces within the laryngotracheal complex. Triggers often include actions such as coughing, sneezing, or violent retching. Given the delicate structures involved in this region, careful assessment is essential to ensure proper management and prevent complications. In this patient’s case, the only reported sequel was the inability to reach high notes while singing.

## Introduction

Spontaneous laryngeal perforation is a rare condition. Non-traumatic fractures of the larynx occur when pressure within the laryngotracheal complex increases with a closed glottis. This event happens during coughing or swallowing, resulting in a significant force on the laryngeal cartilage [1].

An irritative sneeze is an involuntary expulsion of air following an irritating stimulus in the nasal cavity, which stimulates the nerve endings of the maxillary branch of the trigeminal nerve and the palatine nerves, transmitting signals to the medulla oblongata near the respiratory center, where the sneeze control center is located. The respiratory response includes an initial inhalation phase, during which the epiglottis and vocal cords close, and the abdominal expiratory muscles tense, causing an increase in lung pressure. When sufficient pressure builds, the vocal cords relax, the epiglottis opens, and air is forcefully expelled through the nose and mouth, clearing irritants from the nasal cavity. The biomechanical forces generated during a sneeze and their distribution depend on the body’s position, cartilage ossification patterns, and the closed configuration of the glottis, mouth, and nostrils [2].

The only sequela the patient experiences is an inability to reach high notes while singing. A review of the literature revealed no prior documentation of such sequelae, making it an important topic to discuss. Vocal folds are highly elastic, and their histological composition and mechanical properties allow for versatility in voice tones. The tone depends on the length and thickness of each individual's vocal folds, with the vibratory range allowing adjustments to the intensity of the sound produced [3].

## Case presentation

A previously healthy 35-year-old male with a history of seasonal rhinitis presented to the emergency department after sneezing with his mouth closed. Following the sneeze, he experienced spasms, a sensation of obstruction, and crepitus upon touching the anterior neck.

Imaging studies and laryngoscopy

Clinical examination of the neck revealed subcutaneous emphysema. In the absence of a history of trauma, laryngoscopy was recommended. Due to the patient’s significant subjective complaints, the ENT (ear, nose, and throat) team performed a flexible fiberoptic laryngoscopy, which revealed a hematoma in the right vocal cord as the only finding.

**Figure 1:**
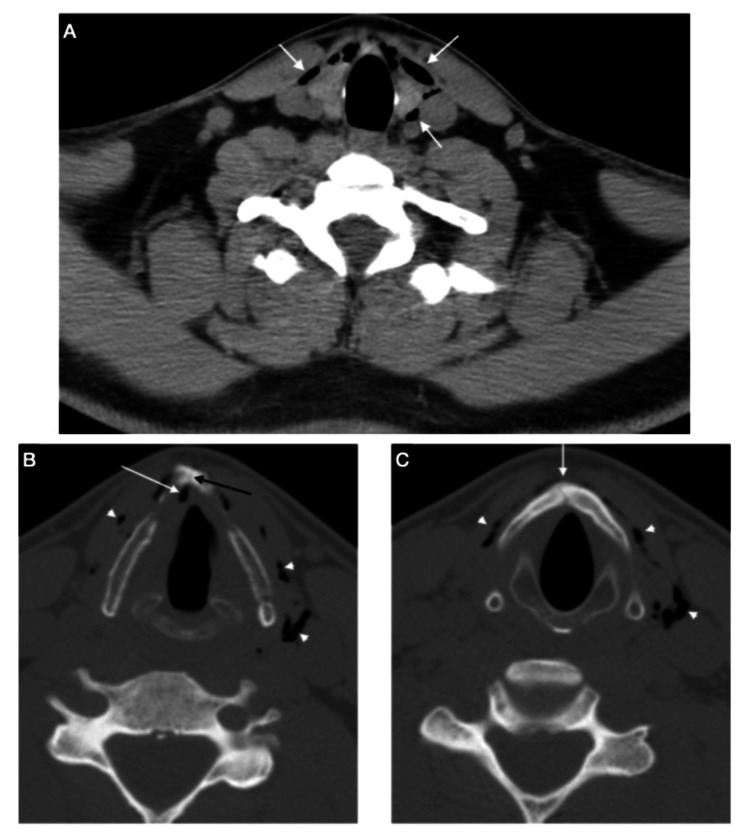
Simple tomography of the neck in soft tissue window (a) and bone window (b, c). (a) Emphysema in the visceral space, with gas extending to the level of the sternohyoid muscles and surrounding the predominantly left-sided thyroid (arrows). (b) Fracture site of the thyroid cartilage adjacent to the vertex (arrow) and emphysema in the visceral space, surrounding the thyroid cartilage (arrowhead). (c) Emphysema adjacent to the insertion site of the right vocal cord and the anterior commissure (arrow). Site of thyroid cartilage fracture (black arrow) and emphysema (arrowheads).

**Figure 2:**
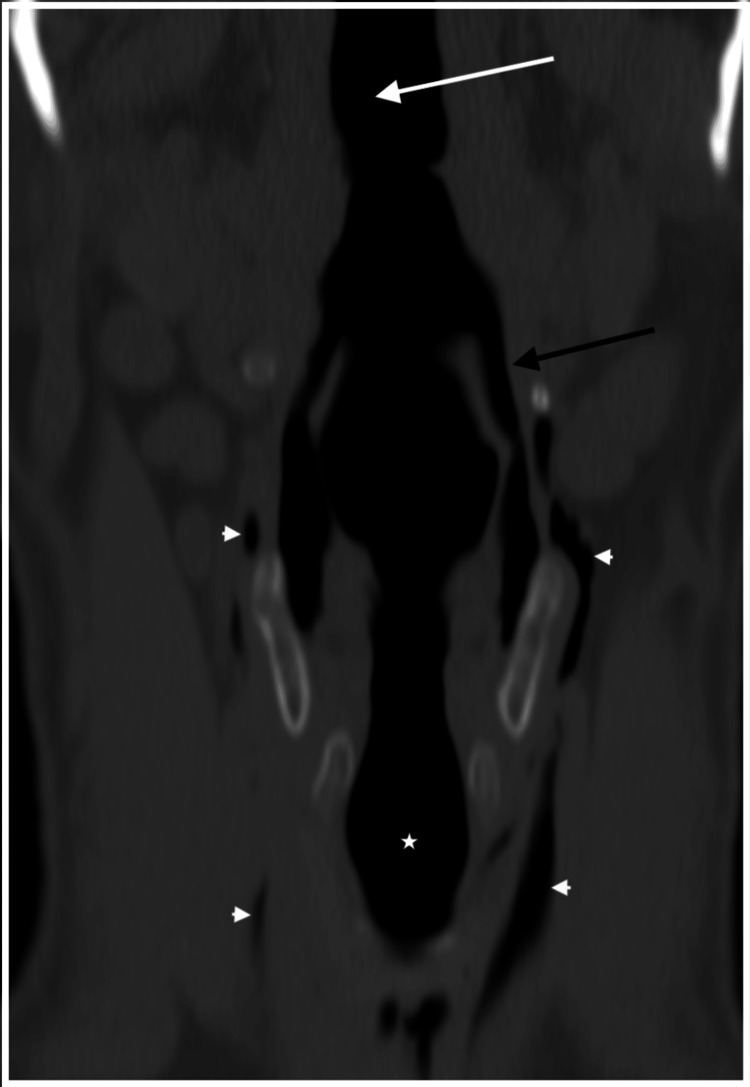
Bone window coronal CT of the neck. Aero-digestive tract (white arrow), proximal trachea (*), pyriform sinus (black arrow), and emphysema in the visceral space (arrowheads).

A non-contrast computed tomography (CT) scan of the neck showed a nondisplaced fracture of the thyroid cartilage adjacent to the vertex (Figures 1B, C), along with emphysema in the visceral space adjacent to the insertion site of the right vocal cord (Figures 1, 2).

Treatment

Diagnostic aids facilitate successful conservative management in cases of non-traumatic laryngeal injury. Securing the airway is the primary goal of early management. The most widely used classification system in the literature for laryngeal injuries is the Schaefer-Fuhrman classification, which assigns injuries a severity scale from 1 to 5. The extent of the injury determines the management approach [4]. Groups 1 and 2 are typically managed conservatively, while higher severity groups usually require surgical repair.

In this patient’s case, the surgical team opted for conservative management since the injury did not compromise the airway or mediastinum. Treatment involved a seven-day course of an antimicrobial regimen (levofloxacin) and steroids (prednisone), along with ten days of vocal rest. Nonsurgical management aims to reduce edema, manage secretions effectively, and prevent inflammation caused by acid reflux to promote recovery of the injured structures.

**Figure 3:**
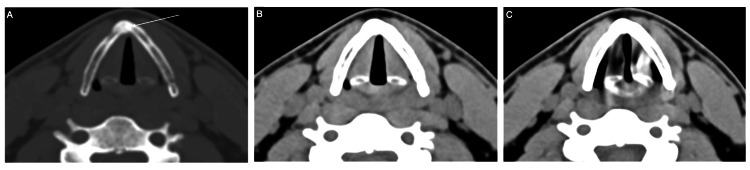
Simple tomography of the neck in bone window (A) and soft tissue window (B, C). Control at eight months post-injury: (a) Formation of repair tissue at the fracture site and resolution of the emphysema (arrow). (b) Comparison at rest with free breathing and during phonation, showing partial abduction of the vocal cords during phonation. (c) No vocal cord paralysis was observed.

**Figure 4:**
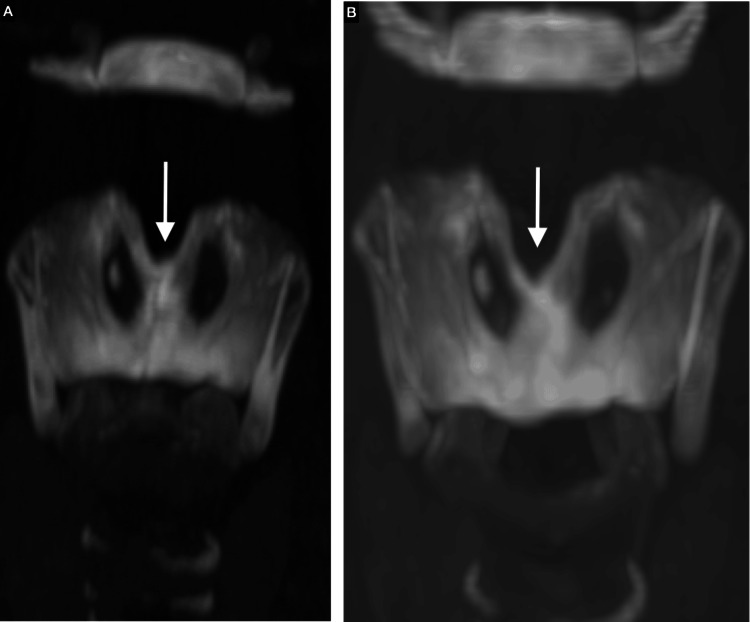
Coronal maximum intensity projection (MIP) at 60 mm. Comparison of the fracture site: (a) initial fracture site and (b) eight months later (white arrow).

At his eight-month follow-up, the patient reported that his only persistent symptom was the inability to reach high notes while singing. A follow-up CT scan of the neck revealed the formation of repair tissue at the fracture site and complete resolution of the emphysema (Figures 3, 4).

## Discussion

Among documented cases, sneezing was the most common cause of thyroid cartilage fractures, followed by coughing and retching, which increase normal pressure by 5 to 24 times. The mechanism involves a sudden increase in intraluminal pressure against closed vocal cords following exertion, such as coughing, retching, or vomiting [5].

A respiratory response occurs with an initial inhalation, during which the epiglottis and vocal cords close, and the expiratory muscles of the abdomen tighten, causing an increase in pressure in the lungs. When sufficient pressure builds, the vocal cords relax, the epiglottis opens, and air escapes through the nose and mouth, allowing the force of the sneeze to expel irritants from the nasal cavity. The pressure of a normal sneeze ranges from 50 to 140 mmHg, at a speed of 70 to 130 km/h. A condition predisposing to spontaneous laryngeal rupture is sneezing with both nostrils and the mouth obstructed. Sound production depends on the movement of the vocal cords. The cartilages of the larynx allow varying degrees of opening between the cords and influence the elevation or depression of the laryngeal structure, thus altering the pitch of the sounds produced by the airflow. The larynx moves: it rises during swallowing and high-pitched sounds and falls during low-pitched sounds [6].

The base of the arytenoid has a lateral prominence: the muscular process, where the posterior cricoarytenoid and anterior cricoarytenoid muscles insert. These muscles rotate the arytenoid cartilage laterally and medially, adducting the vocal ligaments (vocal cords) that vibrate with exhaled air and, along with the mouth's elements (lips, palate, tongue, etc.), produce the voice. The cricoarytenoid articulation is crucial as it allows the vocal folds to move; it connects the base of the arytenoid to the upper edge of the cricoid, enabling the arytenoids to slide, turn, and separate to abduct or adduct the vocal folds. These movements also facilitate rotational movements of the arytenoids in the vertical plane, contributing to abduction or adduction of the vocal folds [7].

The intrinsic or thyroarytenoid muscles run parallel to the vocal cord ligament and account for most of the vocal cord's volume. These muscles divide into two bellies: medial (vocal) and lateral, which run parallel to each other and are responsible for sound emission. The principal muscle is the medial thyroarytenoid, also known as the vocal cord muscle. It constitutes a significant portion of the vocal cord's thickness. The superior thyroarytenoid muscle attaches anteriorly to the upper third of the internal angle of the thyroid cartilage and posteriorly to the muscular process of the arytenoid cartilage. It adducts the vocal cords by engaging the arytenoid cartilage [7].

The cricothyroid muscle and the thyroarytenoid ligament primarily regulate voice pitch. Pitch changes are achieved by modifying the position and tension of the vocal cords. Adjusting vocal cord tension and regulating exhalation force can alter the pitch and intensity of the sound. When the arytenoid cartilages tilt and increase the distance between the thyroid prominences, the cricothyroid muscles tense, raising the pitch of the sound [8].

## Conclusions

Spontaneous laryngeal fractures represent a rare pathology, making a deep understanding of the pathophysiology of the larynx crucial for informed clinical decision-making. Early recognition is of utmost importance to prevent long-term complications.

At tomography, eight months after the injury in our patient, we observed the formation of scar tissue, which lacks the same elastic properties and could affect the ability to achieve high-pitched tones when singing, likely due to an injury to the superior laryngeal nerve. A high level of suspicion is required, emphasizing the need to recognize injury mechanisms and their clinical presentation to ensure early diagnosis and direct appropriate management based on severity.
